# What factors are effective on the CPR duration of patients under extracorporeal cardiopulmonary resuscitation: a single-center retrospective study

**DOI:** 10.1186/s12245-024-00608-2

**Published:** 2024-04-17

**Authors:** Amir Vahedian-Azimi, Ibrahim Fawzy Hassan, Farshid Rahimi-Bashar, Hussam Elmelliti, Anzila Akbar, Ahmed Labib Shehata, Abdulsalam Saif Ibrahim, Ali Ait Hssain

**Affiliations:** 1 Trauma research center, Nursing Faculty, Baqiyatallah University of Medical Sciences, Tehran, Iran; 2 Medical Intensive Care Unit, Hamad General Hospital, Doha, Qatar; 3 Department of Medicine, Weill Cornell Medical College, PO BOX 3050, Doha, Qatar; 4 Department of Anesthesiology and Critical Care, School of medicine, Hamadan University of Medical Sciences, Hamadan, Iran; 5 Emergency Department, Hamad General Hospital, Doha, Qatar; 6 Medical Intensive Care Unit, ECMO team, Hamad General Hospital, Doha, Qatar

**Keywords:** Extracorporeal cardiopulmonary resuscitation, Cardiopulmonary resuscitation, Cardiac arrest, In-hospital cardiac arrest, Out-of-hospital cardiac arrest, Prognostic factors

## Abstract

**Background:**

Extracorporeal cardiopulmonary resuscitation (ECPR) is an alternative method for patients with reversible causes of cardiac arrest (CA) after conventional cardiopulmonary resuscitation (CCPR). However, cardiopulmonary resuscitation (CPR) duration during ECPR can vary due to multiple factors. Healthcare providers need to understand these factors to optimize the resuscitation process and improve outcomes. The aim of this study was to examine the different variables impacting the duration of CPR in patients undergoing ECPR.

**Methods:**

This retrospective, single-center, observational study was conducted on adult patients who underwent ECPR due to in-hospital CA (IHCA) or out-of-hospital CA (OHCA) at Hamad General Hospital (HGH), the tertiary governmental hospital of Qatar, between February 2016 and March 2020. Univariate and multivariate binary logistic regression analyses were performed to identify the prognostic factors associated with CPR duration, including demographic and clinical variables, as well as laboratory tests.

**Results:**

The mean ± standard division age of the 48 participants who underwent ECPR was 41.50 ± 13.15 years, and 75% being male. OHCA and IHCA were reported in 77.1% and 22.9% of the cases, respectively. The multivariate analysis revealed that several factors were significantly associated with an increased CPR duration: higher age (OR: 1.981, 95%CI: 1.021–3.364, *P* = 0.025), SOFA score (OR: 3.389, 95%CI: 1.289–4.911, *P* = 0.013), presence of comorbidities (OR: 3.715, 95%CI: 1.907–5.219, *P* = 0.026), OHCA (OR: 3.715, 95%CI: 1.907–5.219, *P* = 0.026), and prolonged collapse-to-CPR time (OR: 1.446, 95%CI:1.092–3.014, *P* = 0.001). Additionally, the study found that the initial shockable rhythm was inversely associated with the duration of CPR (OR: 0.271, 95%CI: 0.161–0.922, *P* = 0.045). However, no significant associations were found between laboratory tests and CPR duration.

**Conclusion:**

These findings suggest that age, SOFA score, comorbidities, OHCA, collapse-to-CPR time, and initial shockable rhythm are important factors influencing the duration of CPR in patients undergoing ECPR. Understanding these factors can help healthcare providers better predict and manage CPR duration, potentially improving patient outcomes. Further research is warranted to validate these findings and explore additional factors that may impact CPR duration in this population.

## Background

The process of resuscitation following cardiac arrest (CA) has undergone continuous development for over two centuries [1, 2]. CPR (Cardiopulmonary Resuscitation) is an emergency medical procedure performed on individuals who have stopped breathing or whose heart has stopped beating. It involves a combination of chest compressions and rescue breaths to maintain blood circulation and oxygen supply to vital organs until further medical assistance can be provided. Performing CPR immediately after experiencing cardiac arrest significantly increases the chances of survival for the affected individual [3]. However, despite advancements, the mortality rate and neurologic morbidity remains poor in both in-hospital CA (IHCA) and out-of-hospital CA (OHCA) [4, 5]. In general, for every minute that passes without CPR or defibrillation, the survival rate decreases by about 7–10%, and after approximately 10 min without CPR, the chances of successful resuscitation are minimal [6].

The ultimate objective of the resuscitation process is to attain a complete recovery of the patient’s life, ensuring a 100% restoration. Extracorporeal cardiopulmonary resuscitation (ECPR) is an alternative method for patients with CA after conventional CPR (CCPR) [7, 8]. Nonetheless, it is crucial to acknowledge that the survival rate after CPR in cases of CA is influenced by multiple factors [9]. Among these factors, the duration of CPR plays a significant role, as previous studies have demonstrated an inversely associate between the duration of the CPR and the establishment of return of spontaneous circulation (ROSC) [10, 11]. The survival rate and favorable neurological outcomes declines rapidly when the duration of CPR surpasses 10–15 min, with an even faster decline if it exceeds 30 min [12]. Moreover, in case of refractory cardiac arrest, ROSC is not achieved despite more than 30 min of appropriate CPR [13].

Despite well-established protocols and advanced Cardiac Life Support (ACLS) algorithms, determining optimal duration of CPR to optimize the resuscitation process and improve outcomes remains difficult [14, 15]. The duration of CPR after CA can be influenced by various demographic and clinical characteristics such as age [16, 17], comorbidities [18], initial shockable rhythm [19], response time [20], bystander CPR [21], and cause of cardiac arrest [22]. It’s important to note that individual cases may vary, and the outcomes can be influenced by several factors. The decision to continue or terminate CPR depends on several factors, including local protocols, individual patient circumstances, and the medical team’s decision. These factors collectively influence the duration of CPR after cardiac arrest. Unfortunately, very little data exist to guide decision-making in this regard.

Therefore, understanding the factors that can influence the duration of CPR is crucial. This knowledge empowers healthcare professionals to deliver optimal care during cardiac arrest scenarios and make patient-centered decisions that align with their priorities and care goals, ultimately enhancing outcomes. Consequently, this study aimed to examine the different variables impacting the duration of CPR in patients undergoing ECPR.

## Methods

### Study setting and ethical approval

This retrospective, single-center, observational study focused on adult patients who underwent ECPR at Hamad General Hospital (HGH), the tertiary governmental hospital of Qatar, between February 2016 and March 2020. The study strictly adhered to the principles of the Declaration of Helsinki [23], and obtained approval from the Clinical Investigation Ethics Committee of Hamad General Hospital Institutional Review Board (MRC-01-21-934). Informed consent was obtained from all patients or their legal guardians, and the study findings were reported in accordance with the Strengthening the Reporting of Observational Studies in Epidemiology (STROBE) guidelines [24].

### Study subjects

This observational study included a total of 48 consecutive patients who were either presented at Hamad General Hospital (HGH) or directly taken to the hospital after experiencing cardiac arrest. The patients were eligible for enrollment if they were adults and had received resuscitation either by emergency medical services (EMS) following an out-of-hospital cardiac arrest (OHCA) and subsequently transferred to HGH, or if resuscitation had been performed by medical staff after an in-hospital cardiac arrest (IHCA) at HGH, based on the following criteria: (a) age ≥ 18 years, (b) patients underwent ECPR because return of spontaneous circulation (ROSC) could not be achieved through conventional CPR (CCPR). However, patients with unknown arrest time, with a “do not attempt resuscitation” (DNAR) decision, or incomplete registered data were excluded from the study.

### Resuscitation procedure

CPR for OHCA and IHCA was performed by EMS and a resuscitation team in the hospital, respectively, based on the American Heart Association (AHA) 2020 CPR guidelines [25], which included the use of automated external defibrillators, inserting an airway adjunct, establishing a peripheral intravenous line, and administering Ringer’s Lactate solution. In the case of OHCA, EMS providers performed CPR and then transported the patients to the hospital. Chest compressions were continued until ECMO initiation. ECPR was provided to patients who did not achieve return of spontaneous circulation (ROSC) despite at least 20 min of conventional CPR (CCPR), or in cases of unstable vital signs or recurrent CA. The decision to administer ECPR was made by attending physicians in the resuscitation team based on several factors: (a) witnessed arrest, (b) initial electrocardiogram (ECG) findings, (c) presence of a reversible cause of cardiac arrest, (d) call-to-hospital arrival time of ≤ 45 min for OHCA patients, and (e) bystander CPR [26, 27]. However, ECPR was contraindicated in cases of terminal malignancy, severe brain damage, duration exceeding 60 min at the time of initial contact, and age ≥ 80 years [28, 29].

The ECMO team consisted of cardiologists, cardiovascular surgeons, intensivists, special nurses, and perfusionists. In all cases, either the Capiox Emergency Bypass System or the Prolonged Life Support System Cardiohelp ©system (Maquet, Rastatt, Germany), was utilized. To prime the system, a crystalloid solution such as normal saline or a balanced solution was used. No patients received blood-primed ECMO. Initially, a percutaneous vascular approach using the Seldinger technique through the femoral vein and the femoral artery was the technique of choice. The cannula sizes were as follows: Access Cannula: 21–23 FR, Return Cannula: 15–17 FR, Reperfusion Cannula: 7–8 FR [30]. When peripheral cannulation was not possible, central cannulation was performed in the cardiac surgery operating room through the right atrium and the ascending aorta (cannulas used were, respectively, DLP 20–22 Fr and 51 Fr, Medtronic Inc., Minneapolis, MN, USA) [9]. The dimensions of the catheters were selected based on the patient’s body size. The tubing, pump and the oxygenator were all coated with Bioline® coating. Cardiac compression was ceased once successful ECMO pump-on was achieved during CPR. Anticoagulation was achieved by administering a bolus injection of unfractionated heparin, followed by continuous intravenous heparin infusion to maintain an activated clotting time between 160 and 180 s. The initial revolutions per minute of the ECMO device were adjusted to attain an ideal cardiac index greater than 2.6 L/min/m2 of body surface area, central mixed venous oxygen saturation above 70%, and a mean arterial pressure above 65 mm Hg [30]. Blood pressure was continuously monitored through an arterial catheter, and arterial blood gas analysis for estimating cerebral oxygenation was conducted using an artery in the right arm. Additionally, to provide brain protection following cardiac arrest, all patients received hypothermic treatment (32–34 °C) within the first 24 h.

### Data collection

The data for this study were obtained from the medical records of patients who experienced OHCA and IHCA. The records were documented by paramedics, emergency physicians, medical staff in the emergency department (ED) for OHCA patients, and the resuscitation team for IHCA patients. The collected data included baseline demographic and clinical characteristics, such as age, gender, nationality, body mass index (BMI), presence of comorbidities, severity of illness assessed through Acute Physiology and Chronic Health Evaluation (APACHE-II) [31], and Sequential Organ Failure Assessment (SOFA) [32] scores, as well as the cause of cardiac arrest (e.g., ischemic heart disease, pulmonary embolism, or other factors) was collected. Additionally, resuscitation data encompassed details such as the time of collapse, presence of witnesses, performance of bystander CPR, initial shockable rhythm (ventricular fibrillation or pulseless ventricular tachycardia) or non-shockable (pulseless electrical activity or asystole), time intervals in minutes between collapse-to-CPR, and duration of CPR were collected. Moreover, laboratory tests after CA including pH, partial pressure of carbon dioxide (PaCO2), oxygen pressure in the blood (PO2), bicarbonate (HCO3), lactate, international normalized ratio (INR), troponin, and creatinine after CA were recorded and evaluated for all participants.

### Study outcome

The outcome of the study was the duration of CPR, which in this study was measured from the moment of starting CPR (chest compressions) to the time of starting ECPR.

### Statistical analysis

Continuous variables are presented as means ± standard deviation (SD), while categorical variables are shown as numbers and relative frequencies. The baseline demographic and clinical characteristics of CA patients were compared based on CPR duration (≤ 40 min vs. >40 min) using the Student’s t-test or Mann-Whitney tests for continuous variables and the Chi-square or Fisher’s exact tests for categorical variables. Univariate and multivariate binary logistic regression analyses were performed to assess the prognostic value of CPR duration, presenting results as odds ratios (OR) and 95% confidence intervals (95% CI). Variables with a P-value < 0.2 in the univariate analysis were included in the multivariate analysis. Additionally, linear regression was used to evaluate the prognostic value of CPR duration based on laboratory tests. All analyses were conducted using SPSS software (ver.21) (SPSS Inc., Chicago, IL, USA), and a two-tailed P-value of < 0.05 was considered significant. GraphPad Prism 9© (GraphPad Software Inc., La Jolla, CA) was utilized to create a forest plot for the multivariate logistic regression analysis, identifying independent factors associated with the duration of CPR.

## Results

### Study participants characteristics

This study involved a total of 48 participants, with a mean ± SD age of 41.50 ± 13.15 years and 75% of them being male. In terms of nationality, 37.5% were from the Middle-East/Africa region, while 62.5% were from Asian/South Asian countries. The participants had an average body mass index (BMI) of 26.52 ± 5.23. The severity of the disease was assessed using the SOFA and APACHE II scores, which had a mean ± SD value of 11.69 ± 4.12 and 27.54 ± 8.21, respectively. Among the participants, 31.3% had comorbidities, with the most common types being prior ischemic heart disease (18.8%), diabetes (12.5%), hypertension (8.3%), and chronic respiratory disease (16.7%). Regarding the location of CA, 22.9% occurred in the hospital, while 77.1% occurred outside the hospital. Bystander-witnessed events and bystander CPR were reported in 93.8% and 97.9% of cases, respectively. Among the participants, 33.3% experienced an initial shockable rhythm during CA. The mean ± SD time intervals from collapse-to-CPR was 6.31 ± 3.37. The most common cause of cardiac arrest reported in the study was ischemic heart disease (43.8%), followed by pulmonary embolism (12.5%), and other causes (43.8%).

### Demographic and clinical characteristics according to CPR duration

Table 1 presents the baseline demographic and clinical characteristics of study participants based on CPR duration. Several variables showed significant differences. The mean ± SD age (50.32 ± 14.21 vs. 37.10 ± 9.43, *P* = 0.001), BMI (28.98 ± 6.37 vs. 24.92 ± 3.63, *p* = 0.007), SOFA score (15.26 ± 2.28 vs. 9.34 ± 3.27, *P* = 0.001), APACHE II score (30.47 ± 6.76 vs. 25.62 ± 8.61, *P* = 0.044), collapse-to-CPR time (7.89 ± 2.58 vs. 5.28 ± 3.46, *P* = 0.007) were significantly higher in the CPR duration > 40 min group compared to the CPR duration ≤ 40 min group. The percentage of participants with in-hospital cardiac arrest (5.3% vs. 43.5%, *P* = 0.019) and initial shockable rhythm (15.8% vs. 44.8%, *P* = 0.037) was significantly lower in the CPR duration > 40 min group compared to the CPR duration ≤ 40 min group. Furthermore, the percentage of participants with comorbidities (52.6% vs. 17.2%, *P* = 0.01) was significantly higher in the CPR duration > 40 min group compared to the CPR duration ≤ 40 min group. However, there were no significant differences in gender (*P* = 0.233), nationality (*P* = 0.493), types of comorbidities (*P* > 0.05), bystander-witnessed CPR (*P* = 0.819), bystander CPR (*P* = 0.396), and cause of cardiac arrest (*P* = 0.223) based on CPR duration.

**Table 1:** Baseline demographic and clinical characteristics of study participants according to CPR duration

Variables		Total (*n* = 48)	CPR duration		P-value
			≤ 40 min **(***n* **=** **29)**	> 40 min **(***n* **=** **19)**	
Age, (years) #	(Mean ± SD)	41.50 ± 13.15	37.10 ± 9.43	50.32 ± 14.21	0.001*
Gender, n (%) †	Male	36 (75)	20 (69)	16 (84.2)	0.233
	Female	12 (25)	9 (31)	3 (15.8)	
Nationality, n (%) †	Middle-East/Africa	18 (37.5)	12 (41.4)	6 (31.6)	0.493
	Asian/South Asian	30 (62.5)	17 (58.6)	13 (68.4)	
Body Mass Index #	(Mean ± SD)	26.52 ± 5.23	24.92 ± 3.63	28.98 ± 6.37	0.007*
Severity of disease,	SOFA score	11.69 ± 4.12	9.34 ± 3.27	15.26 ± 2.28	0.001*
(Mean ± SD) #	APACHE II score	27.54 ± 8.21	25.62 ± 8.61	30.47 ± 6.76	0.044*
Comorbidities †	yes (%)	15 (31.3)	5 (17.2)	10 (52.6)	0.010*
Types of comorbidities †	Prior Ischemic heart disease	9 (18.8)	3 (10.3)	6 (31.6)	0.127
	Diabetes	6 (12.5)	2 (6.9)	4 (21.1)	0.197
	Hypertension	4 (8.3)	3 (10.3)	1 (5.3)	0.479
	Chronic respiratory disease	8 (16.7)	4 (13.8)	4 (21.1)	0.695
Cardiac arrest location †	In hospital (IHCA)	11 (22.9)	10 (34.5)	1 (5.3)	0.019*
	Out-of-hospital (OHCA)	37 (77.1)	19 (65.5)	18 (94.7)	
Bystander-witnessed †	yes (%)	45 (93.8)	27 (93.1)	18 (94.7)	0.819
Bystander CPR †	yes (%)	47 (97.9)	29 (100)	18 (94.7)	0.396
Initial shockable rhythm †	yes (%)	16 (33.3)	13 (44.8)	3 (15.8)	0.037*
Collapse-to-CPR	(Mean ± SD)	6.31 ± 3.37	5.28 ± 3.46	7.89 ± 2.58	0.007*
Cause of cardiac arrest †	Ischemic heart disease	21 (43.8)	12 (41.4)	9 (47.4)	0.223
	Pulmonary embolism	6 (12.5)	2 (6.9)	4 (21.1)	
	Others	21 (43.8)	15 (51.7)	6 (31.6)	

* *P* < 0.05 considered as significantly, # t-test, † Chi-square test or Fisher exact test, Sequential Organ Failure Assessment (SOFA) Score, Acute Physiology and Chronic Health Evaluation (APACHE II), Cardiopulmonary resuscitation (CPR),

### Laboratory tests according to CPR duration

Analysis of post-cardiac arrest laboratory test results revealed that there were no significant differences in pH, PaCO2, PO2, HCO3, lactate, INR, troponin, and creatinine based on CPR duration. However, trends were observed in certain parameters, such as pH > 7.03 and PaCO2 > 38 mmHg, which showed a higher percentage of patients in the CPR duration > 40 min group (Table 2).

**Table 2:** Post cardiac arrest laboratory tests according to CPR duration

Laboratory tests	Total	CPR duration		P-value
		≤ 40 min **(***n* **=** **29)**	> 40 min **(***n* **=** **19)**	
PH ^a^	7.03 (6.8–7.15)	7.0 (6.8–7.2)	7.12 (6.9–7.2)	0.225
PH ≤ 7.03	23/47 (48.9)	16 (57.1)	7 (36.8)	0.172
PH > 7.03	24/47 (51.1)	12 (42.9)	12 (63.2)	
PaCO2 (mmHg) ^a^	38 (31–47)	40 (32.2–47)	35 (26–46)	0.182
PaCO2 ≤ 38	24/47 (51.1)	13 (46.4)	11 (57.9)	0.440
PaCO2 > 38	23/47 (48.9)	15 (53.6)	8 (42.1)	
PO2 (mmHg) ^b^	301.5 (164.2–4.11)	311 (142-419.5)	292 (167-415-460)	0.850
PO2 ≤ 301	22/44 (50)	12 (48)	10 (52.6)	0.761
PO2 > 301	22/44 (50)	13 (52)	9 (47.4)	
HCO3 (mEq/L) ^c^	10 (7.17–16.3)	9.7 (6.9–16.2)	10 (8.2–17.3)	0.468
HCO3 ≤ 10	25/46 (54.4)	15 (55.6)	10 (52.6)	0.845
HCO3 > 10	21/46 (45.6)	12 (44.4)	9 (47.4)	
Lactate (mmol/L) ^a^	18 (11.5–23)	18.5 (13.6–23.7)	16 (9.6–23)	0.268
Lactate ≤ 18	27/47 (57.5)	14 (50)	13 (68.4)	0.210
Lactate > 18	20/47 (42.5)	14 (50)	6 (31.6)	
INR ^d^	2.2 (1.4–3.8)	3.1 (1.6–5.9)	1.6 (1.4–2.4)	0.057
INR ≤ 2.2	16/31 (51.6)	6 (37.5)	10 (66.7)	0.156
INR > 2.2	15/31 (48.4)	10 (62.5)	5 (33.3)	
Troponin (ng/ml) ^e^	4680 (242-10000)	2061 (152.5-82.93)	5758 (1235.5-11768)	0.145
Troponin ≤ 4680	14/27 (51.9)	8 (61.5)	6 (42.9)	0.332
Troponin > 4680	13/27 (48.2)	5 (38.5)	8 (57.1)	
Creatinine (µmol/L) ^f^	150 (126.5–197)	149 (127–197)	154 (123.7-194.5)	0.864
Creatinine ≤ 150	21/41 (51.2)	13 (56.5)	8 (44.4)	0.443
Creatinine > 150	20/41 (48.8)	10 (43.5)	10 (55.6)	

* *P* < 0.05 considered as significantly, cut-points were presented based on median, Data are presented as median and interquartile ranges (IQR), Data based on; ^a^ 47 patients, ^b^ 44 patients, ^c^ 46 patients, ^d^ 31patients, ^e^ 27 patients and ^f^ 41 patients. partial pressure of carbon dioxide (PCO2), oxygen pressure in the blood (PO2), Bicarbonate (HCO3), International Normalized Ratio (INR). Normal range; PH (7.35 to 7.45), PaCO2 (35 to 42 mmHg), PO2 (80–100 mmHg), HCO3 (22 to 26 milliequivalents per liter), Lactate (2–4 mmol/L), INR (≤ 1.1), Troponin (0 and 0.04 ng/mL) and creatinine (for men: 61.9 to 114.9 µmol/L) and (for women: 53 to 97.2 µmol/L)

### Prognostic CPR duration according to variables

**Fig. 1:**
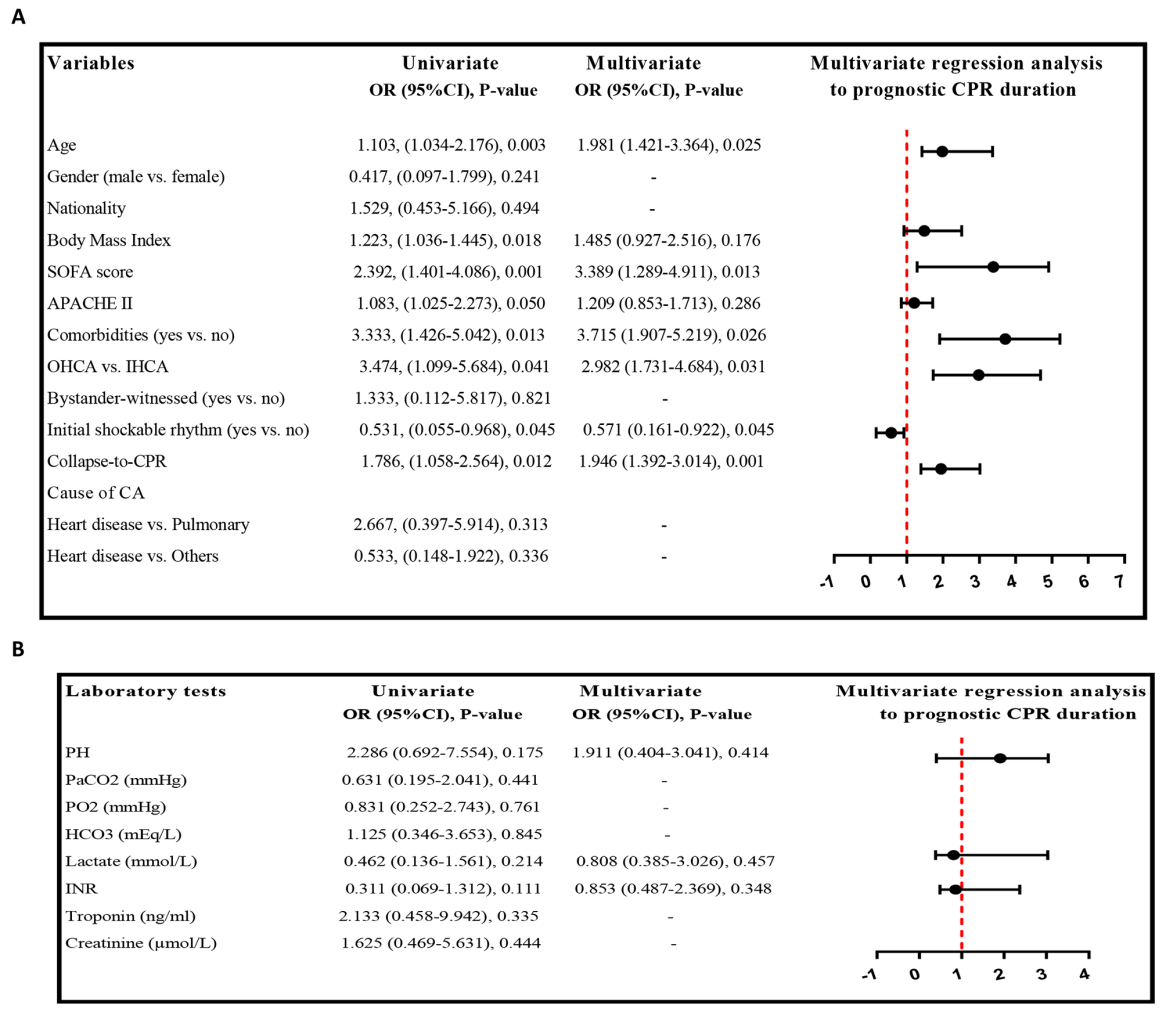
**A** and **BShort title**: Univariate and multivariate binary logistic regression analysis to prognostic CPR duration **Detailed legend**: Univariate and multivariate binary logistic regression analysis to prognostic CPR duration according to (**A**) demographic and clinical variables, and (**B**) laboratory tests. Variables with a P-value < 0.2 in the univariate analysis were included in the multivariate analysis. Abbreviations; Sequential Organ Failure Assessment (SOFA) Score, Acute Physiology and Chronic Health Evaluation (APACHE II), Cardiopulmonary resuscitation (CPR), Extracorporeal cardiopulmonary resuscitation (ECPR), out-of-hospital cardiac arrest (OHCA), in-hospital cardiac arrest (IHCA), partial pressure of carbon dioxide (PCO2), oxygen pressure in the blood (PO2), Bicarbonate (HCO3), International Normalized Ratio (INR). *Nationality (Asian/South Asian vs. Middle-East/Africa).

The results of the univariate and multivariate binary logistic regression analyses revealed significant associations between CPR duration and various demographic and clinical variables. The multivariate analysis revealed that several factors were significantly associated with an increased CPR duration: higher age (OR: 1.981, 95%CI: 1.021–3.364, *P* = 0.025), SOFA score (OR: 3.389, 95%CI: 1.289–4.911, *P* = 0.013), presence of comorbidities (OR: 3.715, 95%CI: 1.907–5.219, *P* = 0.026), OHCA (OR: 3.715, 95%CI: 1.907–5.219, *P* = 0.026), and prolonged collapse-to-CPR time (OR: 1.446, 95%CI:1.092–3.014, *P* = 0.001). Additionally, the study found that the initial shockable rhythm was inversely associated with the duration of CPR (OR: 0.271, 95%CI: 0.161–0.922, *P* = 0.045) (Fig. 1A). However, no significant associations were found between laboratory tests (PH, PCO2, PO2, HCO3, lactate, INR, Troponin, and Creatinine) and CPR duration according to the univariate and multivariate binary logistic regression analysis (Fig. 1B).

### Linear regression findings

The linear regression analysis was conducted to examine the relationship between laboratory tests and the prognostic duration of CPR (Table 3). The results indicate that none of the laboratory tests, including PH, PCO2, PO2, HCO3, Lactate, INR, and Creatinine, show a statistically significant relationship with CPR duration. However, there is a marginally significant positive relationship between Troponin and CPR duration, suggesting that higher Troponin levels may be associated with longer CPR durations.

**Table 3:** Linear regression analysis to prognostic CPR duration according to post cardiac arrest laboratory tests

Laboratory tests	B	Std. Error	Beta	t	P-value
PH	-1.312	15.485	-0.013	-0.085	0.933
PCO2	-0.135	0.187	-0.106	-0.718	0.476
PO2	0.016	0.022	0.117	0.761	0.451
HCO3	-0.392	0.594	-0.099	-0.66	0.513
Lactate	-0.028	0.259	-0.016	-0.107	0.916
INR	-0.162	1.056	-0.029	-0.154	0.879
Troponin	0	0	0.338	1.796	0.085
Creatinine	-0.019	0.051	-0.06	-0.376	0.709

Abbreviations; partial pressure of carbon dioxide (PCO2), oxygen pressure in the blood (PO2), Bicarbonate (HCO3), International Normalized Ratio (INR), B represents the unstandardized coefficient, which indicates the change in the dependent variable (prognostic CPR duration) associated with a one-unit change in the corresponding independent variable (laboratory test). Std. Error refers to the standard error of the coefficient estimate. Beta represents the standardized coefficient, reflecting the standardized effect size of each laboratory test on the CPR duration. t represents the t-value, which tests the significance of the coefficient estimate. P-value indicates the level of statistical significance for each laboratory test’s contribution to the linear regression model

## Discussion

CPR is a lifesaving technique that combines chest compressions and rescue breaths to maintain circulation and oxygenation when the heart stops beating [33]. The duration of CPR plays a crucial role in determining the likelihood of a successful outcome in cardiac arrest patients [34]. Research has consistently demonstrated that shorter CPR duration is associated with higher survival rates and more favorable neurological outcomes [10, 11, 35]. Prognostic factors for CPR duration largely overlap with those for favorable outcomes [36]. Hence, monitoring and optimizing the prognostic factors for the duration of CPR can help to achieve the favorable outcomes [37, 38]. This study highlighted the prognostic factors associated with the duration of CPR in patients undergoing ECPR following IHCA or OHCA. Our findings indicate that age, SOFA score, comorbidities, OHCA, collapse-to-CPR time, were identified as significant predictors of longer CPR duration. Additionally, the initial shockable rhythm was inversely associated with the duration of CPR. However, no significant associations were found between laboratory tests and CPR duration.

Several studies have investigated the prognostic factors influencing CPR duration and favorable outcomes [39–41]. These studies have identified various pre-arrest factors, including age, comorbidities, place of cardiac arrest, and initial shockable rhythm, as well as intra-arrest factors such as location of cardiac arrest and collapse-to-CPR time, associated with CPR duration, which can be helpful in assessing expected prognosis and risk stratification. Consistent with our findings, Hirlekar et al. [42] showed that increasing age and comorbidities (heart failure, stroke, renal dysfunction and history of malignancy) were associated with increase CPR duration and negatively associated with survival following IHCA, even after adjusting for baseline characteristics and intra-arrest factors. A retrospective study by Lee et al. [43], on 111 ECPR patients showed that survival to discharge was associated with a shorter CPR duration, younger age, and a higher rate of initial shockable rhythm. According to a meta-analysis of 23 studies, patient survival following IHCA was found to be inversely correlated with high age, malignancy, comorbidities, and longer CPR duration (> 15 min), while witnessed arrest and initial shockable rhythm were associated with increased survival [40]. A systematic review by Gijn et al. [41], showed that the chances of survival to hospital discharge for in-hospital CPR decreased with age. Data from a prospective registry in British Columbia was analyzed for 2,532 OHCA patients and revealed that 94% of patients with initial shockable rhythms had shorter CPR durations [44]. A meta-analysis of 15 primary studies, which included a total of 841 patients who underwent ECPR following OHCA, revealed a significant association between favorable outcomes and lower CPR duration, initial shockable rhythm, arterial pH, and serum lactate level [45].

Understanding the influence of demographic and clinical characteristics on the duration of CPR after CA is crucial for healthcare providers. This knowledge enables them to make informed decisions about resuscitation efforts, tailoring them to individual patients’ circumstances. Factors such as age, comorbidities, initial rhythm, severity of illness, arrest location (OHCA vs. IHCA), collapse-to-CPR time, and the cause of CA should be considered by medical teams. By doing so, they can assess the likelihood of successful resuscitation and determine the appropriate duration of CPR attempts. This understanding optimizes resource allocation, prevents futile interventions in cases with minimal chances of a positive outcome, and improves overall patient outcomes. However, since demographic characteristics are typically patient-specific and non-modifiable pre-arrest factors, clinicians should exercise caution when using these results to make clinical decisions about starting or stopping resuscitation after cardiac arrest.

### Strengths and limitations

The main strength of this study was investigating a range of demographic, clinical, and laboratory variables, which provided a comprehensive understanding of the factors influencing CPR duration in patients undergoing ECPR. This can help enhance the clinical relevance of the research, as healthcare providers can utilize this knowledge to optimize resuscitation efforts and potentially improve patient outcomes. However, the study had some limitations. Firstly, it was a single-center study, which may restrict the generalizability of the findings to other healthcare settings or populations with different characteristics. Secondly, being a retrospective study, it is subject to inherent limitations such as reliance on existing medical records, potential missing data, and limited control over the quality and completeness of collected data. Additionally, due to the nature of all observational studies, interpretation of the results should be regarded as associative rather than causative. Lastly, the small sample size can influence the accuracy and reliability of the results, potentially leading to conflicting conclusions.

## Conclusion

The findings from the study revealed several significant factors associated with an increased CPR duration. These factors included age, SOFA score, presence of comorbidities, OHCA, and prolonged collapse-to-CPR time. Additionally, the initial shockable rhythm was found to be inversely associated with CPR duration, indicating that patients presenting with shockable rhythms at the onset of CA required shorter durations of CPR. Although laboratory tests were evaluated, no significant associations were found between these parameters and CPR duration. This study provides valuable insights into the factors influencing the duration of CPR in patients undergoing ECPR. The knowledge gained from this study can contribute to improving resuscitation strategies and optimizing outcomes for these critically ill patients.

## Data Availability

The data that support the findings of this study are available from the corresponding author upon reasonable request.

## Abbreviations

CPR: Cardiopulmonary resuscitation
ECPR: Extracorporeal cardiopulmonary resuscitation
ROSC: Return of spontaneous circulation
OHCA: Out-of-hospital cardiac arrest
IHCA: In-hospital cardiac arrest
ECMO: Extracorporeal membrane oxygenation
CA: Cardiac arrest
ACLS: Advanced Cardiac Life Support
EMS: Emergency medical services
APACHE-II: Acute Physiology and Chronic Health Evaluation
SOFA: Sequential Organ Failure Assessment
VV-ECMO: veno-venous ECMO
VA-ECMO: veno-arterial ECMO
OR: Odds ratio
CI: Confidence intervals
